# Management of Primary Dysmenorrhea among University Students in the South of Spain and Family Influence

**DOI:** 10.3390/ijerph17155570

**Published:** 2020-08-01

**Authors:** María Laura Parra-Fernández, María Dolores Onieva-Zafra, Ana Abreu-Sánchez, Juan Diego Ramos-Pichardo, María Teresa Iglesias-López, Elia Fernández-Martínez

**Affiliations:** 1 Department of Nursing, Physiotherapy and Occupational Therapy, University of Castilla-La Mancha, Ciudad Real, 13071 Ciudad Real, Spain; marialaura.parra@uclm.es (M.L.P.-F.); mariadolores.onieva@uclm.es (M.D.O.-Z.); 2 Department of Nursing, University of Huelva, 21071 Huelva, Spain; abreu@denf.uhu.es (A.A.-S.); juan.ramos@denf.uhu.es (J.D.R.-P.); 3 Faculty of Health Sciences, Universidad Francisco de Vitoria, Crta. Pozuelo-Majadahonda Km 1,800, 28223 Pozuelo de Alarcón, Madrid, Spain; m.iglesias.prof@ufv.es

**Keywords:** dysmenorrhea, menstrual pain, self-care

## Abstract

The present study analyses the management of primary dysmenorrhea by university students in the south of Spain. In this cross-sectional observational study, 224 women participated, using an ad hoc self-report questionnaire about menstrual pain and self-care and including sociodemographic and gynecological variables. Some 76.8% of participants consumed analgesics and the majority self-medicated with non-steroidal anti-inflammatory drugs (NSAIDs) without consulting a health professional, with a correlation between pain intensity and the number of pills ingested during menstruation (*r* = 0.151, *p* < 0.05). The higher proportion of women who found their analgesia effective were those who took medication after being prescribed by a health care provider (60.8%) compared to those who self-medicated (40%; *p* < 0.01). Only 43.8% employed non-pharmaceutical methods, most commonly antalgic positions, massages and local heat. These choices were not related to the intensity of menstrual pain nor with the severity of the dysmenorrhea, nor did these most common methods prove to be the most effective. However, a higher percentage of women using non-pharmacological methods was identified in women with family members suffering from dysmenorrhea (73.2%) compared to those without (60%; *p* = 0.040), which may indicate that the choice of remedies is more related to learning self-care in the family context. This study identifies the need for education on self-care and management of menstrual pain.

## 1. Introduction

Primary dysmenorrhea is pain associated with menstruation in which there is no identified organic cause and which is fundamentally associated with an excess of prostaglandins [1,2,3]. This is a periodic and predictable pain that generally occurs just prior to or at the start of menstruation [4]. Primary dysmenorrhea is estimated to affect 50–90% of the world’s young female population [5,6,7,8]. In Spain, there are few studies on this problem; we found only two studies, which estimate the prevalence of dysmenorrhea among the general population to be 56–62% [9,10], while among Spanish university students the prevalence is estimated at approximately 75% [11]. This problem has gained increasing attention and importance as recent studies show that primary dysmenorrhea is associated with diminished quality of life, absenteeism and a drop in academic performance, with a significant socioeconomic impact [8,12,13].

A range of studies show the significant influence of social, cultural and economic factors in dealing with and managing primary dysmenorrhea as identified by Armour et al. in their systematic review and meta-analysis [14]. The majority of studies to date show that a large percentage of women self-medicate and do not consult health care professionals in this matter [11,13,14,15]. The treatment of choice for this problem is generally non-steroidal anti-inflammatory drugs (NSAIDs) [16]. Although most women tend to make the right choice of drug to self-medicate for menstrual pain, this is not usually the case with the dose [5,17,18]. Self-medication with NSAIDs due to their over-the-counter (OTC) status is common in patients suffering from chronic pain like the one presented in this article. However, its handling is not usually successful [17] and poses health risks, including the emergence and aggravation of gastrointestinal problems and adverse drug interactions [19]; therefore, self-medication with NSAIDs for chronic pain is now considered a public health problem [20,21]. Additionally, menstrual pain should not be considered as primary dysmenorrhea until secondary dysmenorrhea has been ruled out by a medical professional and self-medicating pain control is often delayed or dispensed with. In secondary dysmenorrhea, menstrual pain is only a symptom of other gynecological problems, such as endometriosis, and not consulting a doctor may delay diagnosis of these health problems [22,23].

Additionally, there is growing evidence of the beneficial effects of non-pharmacological management of primary dysmenorrhea such as local heat, exercise and acupressure [24,25,26]. This approach is increasingly prevalent due to the absence of any harmful side effects, the ease of this type of self-care and the fact that some 18% of women with dysmenorrhea are resistant to NSAIDs [24,27]. Many authors point to the need for more study of the strategies employed by women to alleviate menstrual pain [12,28]. However, evidence is highly limited regarding the non-pharmacological methods employed by Spanish women to deal with dysmenorrhea while studies in other countries indicate that women do not always choose the most effective strategies for self-management of this problem [12,14,24].

The present study aims to analyze the methods used by Spanish university students to manage their primary dysmenorrhea as well as the perceived effectiveness of the methods employed and compare it between women who have first-degree relatives with dysmenorrhea and those who do not.

## 2. Experimental Section

### 2.1. Design

A cross-sectional observational study conducted in the south of Spain among nursing students of the University of Huelva, Andalusia, between December 2019 and January 2020.

### 2.2. Participants and Sample

The criteria for inclusion in the study were to be between the ages of 18 and 35, enrolled in the Degree in Nursing at the University of Huelva during the 2019/2020 academic year, have dysmenorrhea, not suffer from any diagnosed gynecological pathology (except primary dysmenorrhea) and express a willingness to participate. 

The sample was created by inviting all nursing students enrolled in the 2019/2020 academic year who met these criteria. Of the students 97.8% meeting these criteria agreed to participate in the study.

### 2.3. Study Variables and Data Collection

Data was collected by means of an ad hoc, self-report questionnaire on paper, designed on the basis of previous studies. Participants were invited to take part in the study by a teacher in the classrooms of the Faculty of Nursing and a researcher provided information about the study and was present during the conduct of the study in order to clarify any doubts. The questionnaire consisted of a series of questions about menstrual pain and self-care and including sociodemographic and gynecological variables (Supplementary Materials). The self-reported intensity of menstrual pain was measured using a visual analogue scale (VAS) from 0 to 10 and interpreted as in previous studies: mild (1–3), moderate (4–6) and severe (7–10) [12,29,30,31]. The Andersch and Milsom Scale was used to determine the severity of dysmenorrhea according to series of grades: 0 (menstruation is not painful and daily activity is unaffected), grade 1 (menstruation is painful but seldom inhibits normal activity, analgesics are seldom required and mild pain), grade 2 (daily activity affected, analgesics required and give relief so that absence from work or school is unusual and moderate pain) and grade 3 (activity clearly inhibited, poor effect of analgesics, vegetative symptoms and severe pain) [32,33,34].

### 2.4. Ethics

All students voluntarily participated in the study after signing an informed consent form. The project received a favorable report from the Biomedical Research Ethics Committee of Andalucia (Ref.4/20) was conducted in accordance with the principles of the Helsinki Declaration and all collected data was processed anonymously.

### 2.5. Statistical Analysis

The collected data was loaded onto a Microsoft Office Excel sheet for subsequent analysis using the SPSS v23 statistics package for social sciences (University of Huelva, Huelva, Spain). For the descriptive analysis the frequencies and percentages of the qualitative variables were used while averages and standard deviations were calculated for quantitative variables. 

The chi-square test was used to compare variables at different levels of pain and degrees of dysmenorrhea in different groups, and the unidirectional ANOVA test with a Bonferroni post-hoc test was used on quantitative variables, to compare mean scores in the different groups. An analysis was also made using the Pearson correlation coefficient to analyze the variables of pain intensity using the VAS scale and the number of analgesic pills variables taken per menstrual cycle. The significance level was *p* < 0.05.

## 3. Results

### 3.1. Sociodemographic and Gynecological Characteristics

A total of 224 university students participated in the study, with an average age of 20.96 ± 2.24. Some 75.5% of the women were urban residents compared to 24.5% who were rural residents. The average age of menarche was 12.15 ± 1.52; the majority of the women reported having a regular menstrual cycle (72.8%), with average cycles of 29.05 ± 4.46 days, a menstrual period lasting 5.02 ± 1.26 days and the majority (62.1%) reported experiencing medium flow. Additionally, 65.2% had a first-degree relative, mother or sister, with dysmenorrhea. The pain intensity experienced during the last six months, as rated on the VAS scale, was 6.88 ± 1.78. According to this scale, 6.3% (14) perceived mild pain, 26.3% (59) moderate pain and 67.4% (151) perceived intense pain. According to the Andersch and Milsom scale, of the women with primary dysmenorrhea 0.9% (2) experienced grade 0, 33.9% (79) grade 1, 59.4% (133) grade 2 and 5.8% (13) grade 3.

When grouping the women into three groups based on the intensity of menstrual pain as registered in the VAS, significant statistical differences were detected in the average duration of the menstrual period (*p* < 0.01), identifying a fewer number of days in women with moderate pain; however, there was no correlation between both variables (*r* = 0.079, *p* = 0.242) and no differences were detected in relation to the average duration of the menstrual period when participants were grouped according to their different grades of dysmenorrhea.

Regarding the days when the women experienced menstrual pain, higher average ratings of pain were found for those with a more severe grades of dysmenorrhea in comparison with those with lower grades; a correlation was identified between these two variables (*r* = 0.288, *p* < 0.01). 

Regarding the regularity of the cycle, this was most common in women perceiving mild (71.4%) and moderate (86.4%) pain levels, compared to those experiencing severe pain (67.5%; *p* < 0.05). The consumption of OCP was more frequent in women with lesser grades of dysmenorrhea (*p* < 0.01) and lower intensity of pain according to the VAS scale (*p* < 0.01), as presented in Table 1.

### 3.2. Management of Primary Dysmenorrhea Pain

Only 34.8% of the young women with dysmenorrhea had consulted a health professional about their condition; the proportion was lower among women who had an affected first-degree relative (32.9%) but no statistically significant differences were found when compared to women whose relatives did not have it (38.5%; *p* > 0.05).

Some 76.8% (172) of university students claim to use analgesic drugs to alleviate menstrual pain. A total of 86.8% of students suffering from severe pain take analgesic medication compared to 61% of students suffering from moderate pain and 35.7% of those with mild pain (*p* < 0.01); a correlation was identified between the number of pills ingested and the intensity of menstrual pain using the VAS scale (*r* = 0.151, *p* < 0.05). Average consumption was 3.62 ± 2.74 pills per menstruation, with higher averages found in women suffering from Grade 3 dysmenorrhea (5.15 ± 5.52) compared to those with Grade 2 (3.81 ± 2.45) and Grade 1 (2.42 ± 1.61; *p* < 0.01).

More than half (59.3%) of the women who consumed analgesics for menstrual pain had never consulted a medical professional in this regard and were thus self-medicating. Over half (62.5%) of all the women with primary dysmenorrhea reported the pharmaceuticals used for menstrual pain relief were NSAIDs, followed by paracetamol (19.6%) and other types of non-NSAID analgesics and spasmolytics (8.9%). It should be noted that 27.6% of participants reported self-medicating with a combination of different types of pharmaceuticals. The consumption of NSAIDs was most frequent among women experiencing severe pain (69.5%), compared to those experiencing moderate (52.5%) or mild pain (28.6%; *p* < 0.01). Regarding other types of pharmaceuticals, as presented in Table 2, no significant differences were detected in relation to pain intensity or grade of dysmenorrhea.

The proportion of women taking analgesia for menstrual pain was similar between women who had an affected first-degree relative (76%) and those who did not (78.2%; *p* > 0.05). 

Additionally, some 43.8% (98) of women with primary dysmenorrhea claimed to use non-pharmaceutical methods to alleviate pain, the most common being: antalgic position (23.2%), massages (21.9%), local heat (17.9%) and relaxation (16.1%). There were no differences found regarding the non-pharmacological method chosen and the intensity of the pain or grade of dysmenorrhea as shown in Table 3. However, it was determined that the proportion of women who used non-pharmaceutical methods was higher among those with an immediate family member with dysmenorrhea (73.2%) compared to those who did not (60%; *p* = 0.040). A comparison of each self-care technique identified that analgesic positions were more employed by women with an affected immediate family member (*p* = 0.018).

Among these, some 35.71% (80) reported combining both pharmaceutical and non-pharmaceutical approaches to self-care.

### 3.3. Perceived Effectiveness of Pain Relief

In analyzing the perceived effectiveness of the method used by participants for pain relief from primary dysmenorrhea, findings showed that, with regard to the consumption of pharmaceuticals, 87.8% believed analgesics to be effective in relieving menstrual pain. Of those who consumed NSAIDs, 83.1% reported these analgesics to be effective, 81.8% of those taking paracetamol and 70% of those who consumed other non-NSAIDs analgesics and spasmolytics.

Some 79.6% (78) of participants who used non-pharmaceutical methods reported them to be effective. An analysis of reporting by women using other forms of pain management showed that 100% satisfaction of those using acupressure, acupuncture, aromatherapy, wore a corset, did exercise, had sexual relations, consumed cannabis or meditated; 90% of those using musicotherapy were satisfied; 88.9% of those using relaxation techniques; 87.5% of those using local heat, 77.6% of those using massages; 76.9% of those using antalgic position techniques; 73.3% of those who listened to music and 60% of those who watched television as a means of distraction.

Some 85% of participants who use a combination of pharmaceutical and non-pharmaceutical methods reported this to be effective.

Comparing the perceived efficacy of the use of pharmacological and non-pharmacological methods with the efficacy in women who had and did not have first-degree relatives affected with dysmenorrhea, no differences were found.

A higher percentage of women considered their pharmacological control by means of analgesics effective among those who had consulted a health professional and consumed it on medical advice (60.8%), when compared to those who had not consulted and were self-medicating (40%; *p* < 0.01). Regarding the comparison of satisfaction with non-pharmacological methods in the same groups, although there was also a higher percentage of satisfied women who had consulted a health professional, these differences were not found to be statistically significant.

## 4. Discussion

The present study aimed to analyze the methods of pain relief used by female university students in the south of Spain, specifically Huelva, suffering from primary dysmenorrhea. Some 76.8% of participant consumed analgesics and 43.8% employed non-pharmaceutical methods. With regard to the consumption of pharmaceutical products, it should be noted that a direct correlation was identified between the intensity of pain measured on the VAS scale and the number of pills taken per menstruation (*r* = 0.151, *p* < 0.05). However, more than half of the participants had never consulted a medical professional and were thus self-medicating, most commonly using NSAIDs. The consumption of NSAIDs was most frequent among women suffering from severe dysmenorrhea. Furthermore, the most common non-pharmaceutical methods of pain relief were antalgic positions, massages, local heat and relaxation techniques; the use of these methods does not appear to be related with the intensity of pain nor the grade of dysmenorrhea. Among women who had a first-degree relative with dysmenorrhea, the use of non-pharmacological methods was more common and they consulted health professionals less about it. We also identified a higher percentage of women who considered their method of pain management effective among those who had consulted a health professional about it.

Self-management of pain without consulting a medical professional is highly common, as shown in prior studies in other parts of the world [8,14] and in other regions of Spain [11]. The majority of participants (76.8%) chose pharmaceuticals for relief of menstrual pain compared to only 43.8% who use non-pharmaceutical methods of self-care. The proportion of women who consume analgesics was higher than reported in a recent systematic review and meta-analysis [14] but similar to the findings of prior study conducted in the United States [35]; however, the use of non-pharmaceutical methods was lower than reported in the most prior studies [14,36]. It is worth noting that our study found that the consumption of analgesics, specifically NSAIDs, was much higher in women suffering from severe pain.

It is worrying to have identified a high percentage of women who were self-medicating (59.3%) among future health professionals who will take responsibility for patient safety in the near future. These results are consistent with a previous review that identified variable figures between 21–96% of self-medication in women with dysmenorrhea [8] and others highlight that this type of practice is more frequent in health science students compared to women from other disciplines [37,38]. According to the previous literature, self-medication with OTC and NSAIDs for chronic pain can lead to poor pain management, various side effects including gastrointestinal side effects and rare but serious drug interactions when they occur [17,19,20].

However, our study found no relation between the use of non-pharmaceutical methods and pain intensity or grade of dysmenorrhea, as opposed to the findings of Chen et al., who found greater use of non-pharmaceutical methods among Chinese women the greater their menstrual pain [36]. Findings show that the use of non-pharmaceutical methods was more frequent among women with an immediate family member with dysmenorrhea, which seems to indicate that the choice of these methods is more related to cultural or sociodemographic factors rather than the intensity or grade of dysmenorrhea itself. Earlier studies have found that mothers and sisters are the principal source of information about methods of pain relief for young women with dysmenorrhea [8]. A prior study conducted in Hong Kong found that other variables, apart from pain intensity, such as age, menstrual health education, even the educational level of parents can influence self-care agency and self-care behavior among adolescent girls with dysmenorrhea [39]. Other authors have found that personal beliefs and the capacity for self-control are also factors in the choice of pain management strategies for primary dysmenorrhea [35]. All of these findings point to the need for further study in different contexts and regions.

The results of our study were in line with those of O’Connell et al. and Midilli et al. regarding the most common non-pharmaceutical methods, especially local heat as a method of pain relief [40,41]. Our findings also coincided with the only study of self-care methods among Spanish university students with dysmenorrhea, identifying antalgic positions and local heat as the most frequent method of non-pharmaceutical menstrual pain relief [12]. Compared to studies conducted in other countries, our study found that a lower proportion of women used non-pharmaceutical methods to relieve dysmenorrhea, specifically, a lower number of women used relaxation or distraction techniques such as watching television or listening to music [35,40,42,43]. These differences in the choice of non-pharmaceutical methods may be attributable to educational, economic, ethnic and sociocultural factors as indicated in previous studies [14].

With regard to the perceived effectiveness of analgesics in relieving menstrual pain, our findings show that 83.1% of participants consider NSAIDs to be effective while 16.9% do not. These results are in line with those published by Oladosu et al., which estimated that 18% of women with primary dysmenorrhea are resistant to NSAIDs [27]. However, satisfaction with paracetamol for pain relief was higher than that found in other studies [14], which may encourage further research into other forms of pain management. However, the consumed dose of analgesics was not recorded and taking into account that NSAIDs are sold over the counter in Spain and most women were self-medicating. Therefore, it may be that some women who expressed inefficacy are attributable to the fact that the dose ingested was subtherapeutic as already indicated in a previous study [17].

Furthermore, the results of our study on the perceived effectiveness of non-pharmaceutical methods was similar to the findings of a systematic review published in 2019 by Armour et al. on the use of local heat, exercise and acupressure in pain relief [24]. However, there is no coherence between self-care methods with proven effectiveness and the frequency of their use by the majority of participants in our study, which would appear to point to a significant lack of education in this regard. In our results, it also highlights that the percentage of women who considered their pain management method effective was higher in those who used some non-pharmacological methods (acupressure, acupuncture, aromatherapy, wore a corset, exercise, had sexual relations, consumed cannabis or meditated, musicotherapy, relaxation techniques and local heat) compared to the percentage of women who considered the use of NSAIDs effective. However, non-pharmacological methods are used less than pharmacological ones in our population, probably due to ignorance, poor integration in our society and health field and lack of trust despite the fact that some of them have proven to be effective against this pain and have fewer side effects than drugs. Previous studies have identified that the correct use of nonprescription medicines can be improved by educating consumers in the understanding of labeling and risk assessment [44]. Menstrual pain management could also be improved by implementing educational programs on menstrual pain management and self-medication and its risks aimed at the community that could also be developed in the educational setting from early stages. Previous studies suggest that the correct use of OTC drugs could be improved by educating consumers in understanding labeling and risk assessment.

This study is an initial investigation into the methods of self-care used by university students in the south of Spain in managing with primary dysmenorrhea and the perceived effectiveness of these methods. However, the results should be interpreted with caution given the limitations of the research as a cross-sectional study, evaluating perceived effectiveness through self-reporting and with a sample limited to a single university and faculty. The design is cross-sectional and having included only nursing students limits the possibility of generalizing the results. Being a student nurse and receiving training in various related subjects, such as pharmacology and gynecology, is likely to influence the treatment of dysmenorrhea and the ability to self-medicate. There is also a participation bias in being future health professionals who suffer from a health-related problem, all of which may have led to an overestimation of drug consumption. In the near future, it is planned to carry out a multi-centre study involving students from different disciplines in both health and other sciences, which would allow the results to be compared. It would also be interesting if the study were a longitudinal one on health sciences students to evaluate the impact of university training and life from the time they enter to the time they complete their studies.

On the other hand, it would be interesting to continue in this line of research by exploring the reasons why women choose their method of pain self-management, to analyze their knowledge of pharmacological and non-pharmacological methods for the management of their dysmenorrhea, and to explore why many of them do not consult a health professional.

## 5. Conclusions

Spanish university students with primary dysmenorrhea do not generally consult a healthcare professional and the majority chose to self-medicate to manage their menstrual pain. Less than half use non-pharmacological methods of pain relief and their preferences are not related either to the intensity of menstrual pain or to the proven effectiveness of these methods. Thus, the choice of pain relief methods seems to be subject to educational and cultural factors, influenced mainly by the family context. Women with a first-degree relative with dysmenorrhea use more non-pharmacological methods and consult fewer health professionals about them. Healthcare professionals can promote safer and more effective self-care among women through health education programs aimed at families. It would be interesting for future studies to design, implement and evaluate the impact of a health education program aimed at the community and families to inform them about the most effective methods of self-care and pain relief.

### Implications for Practice

The large percentage of young Spanish women who self-medicate to treat menstrual pain and the limited use of non-pharmacological methods of proven effectiveness when compared to other countries is a demonstration of the educational deficit in this matter and points to the need for education about self-care and the management of menstrual pain among these women. Furthermore, due to the family influence on this care, there is a need for interventions aimed at the community in the same way.

## Figures and Tables

**Table 1:** Comparison of sociodemographic and gynecological factors between women with different levels of menstrual pain and grades of dysmenorrhea.

Sociodemographic and Gynecological Variables		Pain Intensity VAS				Grade of Dysmenorrhea					
		Mild (n = 14) n (%)/M ± DS	Moderate (n = 59) n (%)/M ± DS	Severe (n = 151) n (%)/M ± DS	*p*-Value	0 (n = 2) n (%)/M ± DS	1 (n = 76) n (%)/M ± DS	2 (n = 133) n (%)/M ± DS	3 (n = 13) n (%)/M ± DS	Total n (%)/M ± DS	*p*-Value
Age		20.29 ± 1.77	20.83 ± 1.78	21.07 ± 2.42	0.397 ^a^	21.50 ± 2.12	20.79 ± 1.87	21.02 ± 2.47	21.31 ± 1.84	20.96 ± 2.24	0.813 ^a^
Setting	Urban	13 (7.7%)	43 (25.6%)	112 (66.7%)	0.276 ^b^	1 (0.6%)	56 (33.3%)	101 (60.1%)	10 (6%)	168 (75%)	0.843 ^b^
	Rural	1 (1.8)	16 (28.6%)	39 (69.6%)		1 (1.8%)	20 (35.7%)	32 (57.1%)	3 (5.4%)	56 (25%)	
BMI		20.63 ± 1.38	22.33 ± 3.18	22.47 ± 3.37	0.131 ^a^	25.26 ± 2.73	22.16 ± 2.90	22.41 ± 3.52	21.77 ± 2.34	22.31 ± 3.25	0.528 ^a^
Age at menarche		12.5 ± 1.51	12.27 ± 1.42	12.07 ± 1.55	0.455 ^a^	13.00 ± 1.41	12.09 ± 1.49	12.24 ± 1.56	11.46 ± 1.13	12.15 ± 1.52	0.275 ^a^
Duration of menstrual cycle		28.57 ± 4.27	28.64 ± 3.34	29.25 ± 4.85	0.621 ^a^	28.50 ± 6.36	28.07 ± 3.70	29.55 ± 4.81	29.62 ± 4.29	29.04 ± 4.47	0.137 ^a^
Duration of period		5.43 ± 1.16	4.61 ± 1.16	5.15 ± 1.27	0.008 ^a,^*	3.00 ± 0.00	4.95 ± 1.10	5.08 ± 1.30	5.31 ± 1.55	5.02 ± 1.26	0.096 ^a^
Days of pain		2.08 ± 0.49	2.10 ± 0.78	2.45 ± 1.16	0.057 ^a^	1.50 ± 0.71	2.09 ± 0.83	2.36 ± 1.00	3.69 ± 1.65	2.34 ± 10.6	0.000 ^a,^*
Flow	Light	2 (6.7%)	10 (33.3%)	18 (60%)	0.048 ^b,^*	0 (0%)	14 (46.7%)	16 (53.3%)	0 (0%)	30 (13.5%)	0.183 ^b^
	Medium	9 (6.5%)	43 (30.9%)	87 (62.6%)		2 (1.4%)	49 (35.5%)	78 (56.5%)	9 (6.5%)	138 (61.9%)	
	Heavy	3 (5.5%)	6 (10.9%)	46 (83.6%)		0 (0%)	12 (21.8%)	39 (70.9%)	4 (7.3%)	55 (24.7%)	
Regular	No	4 (6.6%)	8 (13.1%)	49 (80.3%)	0.022 ^b,^*	1 (1.6%)	9 (14.8%)	46 (75.4%)	5 (8.2%)	61 (27.2%)	0.003 ^b,^*
	Yes	10 (6.1%)	51 (31.3%)	102 (62.6%)		1 (0.6%)	67 (41.1%)	87 (53.4%)	8 (4.9%)	163 (72.8%)	
OCP	No	7 (4.1%)	40 (23.3%)	125 (72.7%)	0.003 ^b,^*	1 (0.6%)	48 (27.9%)	113 (65.7%)	10 (5.8%)	172 (76.8%)	0.003 ^b,^*
	Yes	7 (13.5%)	19 (36.5%)	26 (50%)		1 (1.9%)	28 (53.8%)	20 (38.5%)	3 (5.8%)	52 (23.3%)	

^a^ one-way ANOVA test, ^b^ Chicuadrado test, * *p* < 0.05.

**Table 2:** Method of pain relief chosen by women with dysmenorrhea according to pain intensity (VAS) and grade of dysmenorrhea (Andersch and Milsom scale).

Method of Pain Relief		Pain Intensity VAS				Grade of Dysmenorrhea					
		Mild n (%)	Moderate n (%)	Severe n (%)	*p*-Value ^a^	0 n (%)	1 n (%)	2 n (%)	3 n (%)	Total	*p*-Value ^a^
Pharmaceutical Method	No	9 (17.3%)	23 (44.2%)	20 (38.5%)	0.000 *	2 (3.8%)	39 (75%)	11 (21.2%)	0 (0%)	52 (23.3%)	0.000 *
	Yes	5 (2.9%)	36 (20.9%)	131 (76.2%)		0 (0%)	37 (21.5%)	122 (70.9%)	13 (7.6%)	172 (76.8%)	
Non-Pharmaceutical Method	No	9 (7.1%)	34 (27%)	83 (65.9%)	0.773	2 (1.6%)	50 (39.7%)	69 (54.8%)	5 (4%)	126 (56.3%)	0.070
	Yes	5 (5.1%)	25 (25.5%)	68 (69.4%)		0 (0%)	26 (26.5%)	64 (65.3%)	8 (8.2%)	98 (43.8%)	

^a^ Chicuadrado test, * *p* < 0.05.

**Table 3:** Method of pain management chosen by women with dysmenorrhea according to pain intensity (VAS) and grade of dysmenorrhea (Andersch and Milsom scale).

Method of Pain Management		Pain Intensity VAS				Grade of Dysmenorrhea					
		Mild n (%)	Moderate n (%)	Severe n (%)	*p*-Value ^a^	0 n (%)	1 n (%)	2 n (%)	3 n (%)	Total n (%)	*p*-Value ^a^
Acupressure	No	14 (6.3%)	59 (26.7%)	148 (67%)	0.480	2 (0.9%)	76 (34.4%)	130 (58.8%)	13 (5.9%)	221 (98.7%)	0.556
	Yes	0 (0%)	0 (0%)	3 (2%)		0 (0%)	0 (0%)	3 (2.3%)	0 (0%)	3 (1.3%)	
Acupuncture	No	14 (6.3%)	59 (26.5%)	150 (67.3%)	0.784	2 (0.9%)	76 (34.1%)	132 (59.2%)	13 (5.8%)	223 (99.6%)	0.876
	Yes	0 (0%)	0 (0%)	1 (0.7%)		0 (0%)	0 (0%)	1 (0.8%)	0 (0%)	1 (0.4%)	
Relaxation	No	13 (6.9%)	49 (26.1%)	126 (67%)	0.642	2 (1.1%)	64 (34%)	111 (59%)	11 (5.9%)	188 (83.9%)	0.937
	Yes	1 (7.1%)	10 (27.8%)	25 (69.4%)		0 (0%)	12 (33.3%)	22 (61.1%)	2 (5.6%)	3 (16.1%)	
Antalgic Position	No	11 (6.4%)	47 (27.3%)	114 (66.3%)	0.803	2 (1.2%)	64 (37.2%)	98 (57%)	8 (4.7%)	172 (76.8%)	0.147
	Yes	3 (5.8%)	12 (23.1%)	37 (71.2%)		0 (0%)	12 (23.1%)	35 (67.3%)	5 (9.6%)	52 (23.2%)	
Massages	No	12 (6.9%)	51 (29.1%)	112 (64.0%)	0.120	2 (1.1%)	67 (38.3%)	98 (56%)	8 (4.6%)	175 (78.1%)	0.034 *
	Yes	2 (4.1%)	8 (16.3%)	39 (79.6%)		0 (0%)	9 (18.4%)	35 (71.4%)	5 (10.2%)	49 (21.9%)	
Musicotherapy	No	14 (6.5%)	56 (26.2%)	144 (67.3%)	0.698	2 (09%)	73 (31.4%)	126 (58.9%)	13 (6.1%)	214 (95.5%)	0.814
	Yes	0 (0%)	3 (30%)	7 (70%)		0 (0%)	3 (30%)	7 (70%)	0 (0%)	10 (4.5%)	
Watch TV	No	13 (6.2%)	57 (27.3%)	139 (66.5%)	0.493	2 (1%)	71 (34%)	124 (59.3%)	12 (5.7%)	209 (93.3%)	0.983
	Yes	1 (6.7%)	2 (13.3%)	12 (80%)		0 (0%)	5 (33.3%)	9 (60%)	1 (6.7%)	15 (6.7%)	
Listen to Music	No	13 (6.2%)	57 (27.3%)	139 (66.5%)	0.493	2 (1%)	66 (34%)	114 (58.8%)	12 (6.2%)	194 (86.6%)	0.857
	Yes	1 (6.7%)	2 (13.3%)	12 (7.9%)		0 (0%)	10 (33.3%)	19 (63.3%)	1 (3.3%)	30 (13.4%)	
Aromatherapy	No	14 (6.4%)	57 (25.9%)	149 (67.7%)	0.521	2 (0.9%)	73 (33.2%)	132 (60%)	13 (5.9%)	220 (98.2%)	0.375
	Yes	0 (0%)	2 (50%)	2 (50%)		0 (0%)	3 (75%)	1 (25%)	0 (0%)	4 (1.8%)	
TENS	No	14 (6.3%)	59 (26.3%)	151 (67.4%)	-	2 (0.9%)	76 (33.9%)	133 (59.4%)	13 (5.8%)	224 (100%)	-
	Yes	0 (0%)	0 (0%)	0 (0%)		0 (0%)	0 (0%)	0 (0%)	0 (0%)	0 (0%)	
Local Heat	No	11 (6%)	49 (26.6%)	124 (67.4%)	0.925	2 (1.1%)	64 (34.8%)	109 (59.2%)	9 (4.9%)	184 (82.1%)	0.544
	Yes	3 (7.5%)	10 (25%)	27 (67.5%)		0 (0%)	12 (30%)	24 (60%)	10 (30.8%)	40 (17.9%)	
Consume Alcohol	No	14 (6.5%)	57 (26.5%)	144 (67%)	0.672	2 (0.9%)	74 (34.4%)	128 (59.5%)	11 (5.1%)	215 (96%)	0.184
	Yes	0 (0%)	2 (22.2%)	7 (77.8%)		0 (0%)	2 (22.2%)	5 (55.6%)	2 (22.2%)	9 (4%)	
Cannabis	No	14 (6.3%)	58 (26.0%)	151 (67.4%)	0.245	2 (0.9%)	76 (34.1%)	132 (59.2%)	13 (5.8%)	223 (99.6%)	0.876
	Yes	0 (0%)	1 (100%)	0 (0%)		0 (0%)	0 (0%)	1 (100%)	0 (0%)	1 (0.4%)	
Breathing Exercises	No	14 (6.3%)	59 (26.5%)	150 (67.3%)	0.784	2 (0.9%)	76 (34.1%)	132 (59.2%)	13 (5.8%)	223 (99.6%)	0.876
	Yes	0 (0%)	0 (0%)	1 (100%)		0 (0%)	0 (0%)	1 (100%)	0 (0%)	1 (0.4%)	
Evening Primrose Oil	No	14 (6.3%)	59 (26.3%)	151 (67.4%)	-	2 (0.9%)	76 (33.9%)	133 (59.4%)	13 (5.8%)	224 (100%)	-
	Yes	0 (0%)	0 (0%)	0 (0%)		0 (0%)	0 (0%)	0 (0%)	0 (0%)	0 (0%)	
Corset	No	14 (6.3%)	58 (26.0%)	151 (67.4%)	0.245	2 (0.9%)	76 (33.9%)	132 (59.2%)	13 (5.8%)	223 (99.6%)	0.876
	Yes	0 (0%)	1 (100%)	0 (0%)		0 (0%)	0 (0%)	1 (100%)	0 (0%)	1 (0.4%)	
Meditation	No	14 (6.3%)	59 (26.5%)	150 (67.3%)	0.245	2 (0.9%)	75 (33.6%)	133 (59.4%)	13 (5.8%)	223 (99.6%)	0.582
	Yes	0 (0%)	0 (0%)	1 (100%)		0 (0%)	1 (100%)	0 (0%)	0 (0%)	1 (0.4%)	
Exercise	No	13 (5.8%)	59 (26.3%)	151 (67.4%)	0.001 *	2 (0.9%)	75 (33.6%)	133 (59.4%)	13 (5.8%)	223 (99.6%)	0.582
	Yes	1 (100%)	0 (0%)	0 (0%)		0 (0%)	1 (100%)	0 (0%)	0 (0%)	1 (0.4%)	
Sexual Relations	No	13 (5.8%)	59 (26.3%)	151 (67.4%)	0.001 *	2 (0.9%)	75 (33.6%)	133 (59.4%)	13 (5.8%)	223 (99.6%)	0.582
	Yes	1 (100%)	0 (0%)	0 (0%)		0 (0%)	1 (100%)	0 (0%)	0 (0%)	1 (0.4%)	

^a^ Chicuadrado test, * *p* < 0.05, (TENS): Transcutaneus Electrical Nervious Stimulation.

## Supplementary Materials

The following are available online at https://www.mdpi.com/1660-4601/17/15/5570/s1, Questionnaire Q1: Menstrual Pain Management Questionnaire.
