# Supplementary material for: Management of Primary Dysmenorrhea among University Students in the South of Spain and Family Influence
Source: Int J Environ Res Public Health. 2020 Aug 1;17(15):5570. doi: 10.3390/ijerph17155570 (PMC7432638; doi:10.3390/ijerph17155570)
Supplement: Supplementary file 1 [file ijerph-17-05570-s001.pdf]

**Questionnaire**

1. Age:....years
2. Weight: ....kg
3. Height: ....cm
4. Zone of residence: urban/rural
5. Do you have a first degree relative (mother or sister) with dysmenorrhea? yes/no
6. Age of menarche: .......years
7. Have you ever consulted a health care professional about your menstrual pain?yes/no

   Answer the following questions about your menstrual cycles in the last 3 months:

8. Regular menstrual cycle: yes/no
9. Duration of menstrual spotting (average number of days): .....days
10. Amount of menstrual bleeding: light (≤ 5 pads/day)/medium (5-7 pads/day)/heavy (≥ 7 pads/day)
11. Days between periods (average number of days): ....days
12. Do you take hormonal contraceptives?yes/no
13. Do you take any drugs to relieve your menstrual pain? Yes/no
    -Did any health professionals recommend her to you? Yes/no
    -What drug or drugs do you take?............................
    -¿ How many average tablets per period do you take? ................................
    -Do you consider this form of pain relief to be effective? Yes/no
    (if you use several methods please indicate below your opinion on the effectiveness (yes/no) for each of them)
    ......................................................................................................................
    ......................................................................................................................
    Do you use any non-pharmacological methods to relieve your pain? Yes/No
    - What method or methods do you use?.................................................
    -Do you consider this form of pain relief to be effective? Yes/no
    (if you use several methods please indicate below your opinion on the effectiveness (yes/no) for each of them)
    ......................................................................................................................
    ......................................................................................................................

ANEXO: Visual Analogue Scale (VAS) from 0 to 10 adn The Andersch and Milsom Scale were attached as an annex to the questionnaire.
